# Cloud/VPN-Based Remote Control of a Modular Production System Assisted by a Mobile Cyber–Physical Robotic System—Digital Twin Approach

**DOI:** 10.3390/s25020591

**Published:** 2025-01-20

**Authors:** Georgian Simion, Adrian Filipescu, Dan Ionescu, Adriana Filipescu

**Affiliations:** 1 Department of Automation, “Dunarea de Jos” University of Galati, 800008 Galati, Romania; georgian.simion@ugal.ro (G.S.); dan.ionescu@ugal.ro (D.I.); 2 Doctoral School of Fundamental Sciences and Engineering, “Dunarea de Jos” University of Galati, 800008 Galati, Romania

**Keywords:** MPS, MCPRS, WMR, RM, MVSS, SHPN, DT, AR, VR, Profibus, Profinet, Ethernet

## Abstract

This paper deals with a “digital twin” (DT) approach for processing, reprocessing, and scrapping (P/R/S) technology running on a modular production system (MPS) assisted by a mobile cyber–physical robotic system (MCPRS). The main hardware architecture consists of four line-shaped workstations (WSs), a wheeled mobile robot (WMR) equipped with a robotic manipulator (RM) and a mobile visual servoing system (MVSS) mounted on the end effector. The system architecture integrates a hierarchical control system where each of the four WSs, in the MPS, is controlled by a Programable Logic Controller (PLC), all connected via Profibus DP to a central PLC. In addition to the connection via Profibus of the four PLCs, related to the WSs, to the main PLC, there are also the connections of other devices to the local networks, LAN Profinet and LAN Ethernet. There are the connections to the Internet, Cloud and Virtual Private Network (VPN) via WAN Ethernet by open platform communication unified architecture (OPC-UA). The overall system follows a DT approach that enables task planning through augmented reality (AR) and uses virtual reality (VR) for visualization through Synchronized Hybrid Petri Net (SHPN) simulation. Timed Petri Nets (TPNs) are used to control the processes within the MPS’s workstations. Continuous Petri Nets (CPNs) handle the movement of the MCPRS. Task planning in AR enables users to interact with the system in real time using AR technology to visualize and plan tasks. SHPN in VR is a combination of TPNs and CPNs used in the virtual representation of the system to synchronize tasks between the MPS and MCPRS. The workpiece (WP) visits stations successively as it is moved along the line for processing. If the processed WP does not pass the quality test, it is taken from the last WS and is transported, by MCPRS, to the first WS where it will be considered for reprocessing or scrapping.

## 1. Introduction

A cloud and VPN-based remote-control system for a modular production system (MPS), consisting of four workstations that handle processes such as buffering, processing, sorting/storage, reprocessing, and scrapping, is presented in this paper. This system is assisted by a MCPRS [1,2,3]. The DT approach, in this system, integrates both physical and virtual components to enhance process monitoring, task planning, and overall system control. The system involves a MPS with four WSs, arranged in a line-shaped configuration and a MCPRS consisting of two driving wheels/one free wheel (2DW/1FW) WMR, a seven degrees of freedom (DOF) RM, and a MVSS with a camera on the RM’s end effector. This architecture enables the processing, reprocessing, or scrapping of WPs as they move through the production line, using both real-world hardware and a virtual twin for optimized performance. DT integration consists of a combination of AR for task planning and VR for real-time visualization and simulation. The AR for task planning uses Node-RED functions to facilitate user interaction, allowing remote tasks to be setup and validated through an AR interface. VR and simulation use SHPN via the Sirphyco package for dynamic simulations, which include both TPN for the fixed MPS’s WSs and CPN for MCPRS movement control.

The main system objectives are as follows:

- Processing: WP is processed sequentially at stations such as buffering, handling, processing, and sorting/storage.
- Reprocessing: WP failing the Primary Quality Test (PQT) at the sorting and storage WS is transported back by the MCPRS to the buffer WS. This WP undergoes a second cycle of processing to meet quality standards.
- Scrapping: WP failing the Secondary Quality Test (SQT) at the handling WS is classified as scrap. MCPRS ensures this defective WP is segregated for disposal, maintaining production flow efficiency.
- Remote Accessibility: Cloud/VPN-based interfaces provide real-time monitoring and control. The user can observe, plan, and adjust tasks remotely, ensuring flexible and scalable production The user interacts visually with production systems via AR-enhanced Human–Machine Interfaces (HMIs). AR facilitates the real-time visualization of robotic and WS operations.

Although the remote control of P/R/S technology is designed to work on a laboratory structure, MPS assisted by MCPRS has a counterpart in the real world, especially in industries where the recovery and reuse of components is one of the objectives. The most eloquent correspondents in the real world are the manufacturing processes in the automotive industry, car body, gearbox, and engine block manufacturing. In most cases, robotic manipulators that have a fixed location serve these manufacturing lines. Through this study, we extended the degree of automation and efficiency of these production lines using MCPRSs. Across industries, we are seeing a greater use of DT in manufacturing. DT visualization technology pairs well with the sensors that manufacturers use to gather vital information on production processes. Now, the information collected via smart manufacturing systems can be incorporated into visual, interactive models.

MCPRS find applicability in multiple areas like mobile intelligent robots, manufacturing robotic systems, educational mobile devices, intelligent service, and IoT-cloud control. MCPRS could improve efficiency and scalability when processing complex tasks that are impossible under local resource constraints in different applications [1,2]. The MCPRS functions as a mobile cyber–physical system, intertwining hardware and software for adaptive, real-time operations. The system adjusts dynamically to context changes, ensuring robust and efficient performance, and deeply connects computational algorithms and physical processes to ensure synchronization. MCPRS can operate at different spatial and temporal scales, exhibiting multiple and distinct behavioral modalities, and interacting with each other in context-changing ways [1,2,3,4,5,6,7,8,9,10].

DT in manufacturing, also known as a digital replica, is a virtual copy of a real-world component in the manufacturing process. The DT approach enables the real-time digital representation of the entire system, ensuring synchronization between the physical and virtual components.

The virtual counterpart includes AR for task planning and SHPN model simulation as VR. Task planning is enhanced with AR technology, allowing users to interact with the production system visually. Through Node-RED functions, users can visualize real-time processes and plan or modify tasks by interacting with virtual WSs and robotic systems. Users can observe how the MCPRS moves, picks, and places WP, or how each WS handles its respective processing tasks. They can also simulate workflow changes to predict performance. VR by SHPN model simulation combines TPNs and CPNs in a virtual environment, representing the MPS and MCPRS’s movement together. The MPS’s workstations are controlled by TPN, which models the discrete processes at each station. These include WP’s loading, buffing, handling, transferring between stations, main processing operations, and sorting and evaluating quality. The MCPRS’s movement is governed by CPN, representing the dynamic displacement and operation of the WMR, RM, and MVSS [11,12,13,14,15,16,17,18].

The real world consists of communication, synchronization, monitoring, and control of a processing technology with product recovery by reprocessing that works on a laboratory system and integrates several subsystems, namely 4-WS FESTO MPS-200 mechatronics line assisted by MCPRS that consists of an autonomous robotic system, PeopleBot WMR, equipped with a 7-DOF Cyton1500 RM and an MVSS located on the end effector with a camera. All these subsystems are equipped with PLCs, wired and wireless communication devices, infrared, inductive, and optical sensors, and electric and pneumatic actuators.

The technology assisted by MCPRS is a type of pipeline, that allows processing operations, quality tests, reprocessing with the same operations, or complete rejection if the WP is completely compromised [18,19,20,21,22,23,24,25,26,27]. A combination of TPNs and CTNs is used in the virtual representation of the system to synchronize tasks between the MPS and MCPRS. This enables the coordination of discrete-event processes with continuous movement in a unified framework.

A SCADA system is integrated into the MPS-MCPRS setup for real-time control and automation, gathering data from IoT sensors, actuators, and other connected devices to provide continuous feedback to the user. The system enables users to monitor and control the MCPRS’s movements, the sequencing of MPS processing operations, and the status of all connected devices. SCADA interfaces offer real-time visualizations, displaying key information such as WP positions, actuator states, and task progression, allowing users to make informed decisions based on live system data.

Two HMIs are implemented for both local and remote interaction:

- HMI-MPS: Dedicated to monitoring and controlling the MPS.
- HMI-MCPRS: Focused on managing MCPRS operations.

These HMIs provide real-time feedback from SCADA, DT, and IoT sensors, offering detailed information on the P/R/S progress of each WP and enhancing operational clarity and decision-making.

OPC-UA is integrated to ensure standardized and secure communication between the various devices within the MPS assisted by MCPRS. This facilitates seamless interoperability among the MPS WSs, MCPRS subsystems (WMR, RM, and MVSS), IoT sensors, PLCs, embedded computers, IoT gateways, and cloud platforms. Acting as a bridge between local control systems (SCADA and HMIs) and the cloud-based infrastructure, OPC-UA, ensures smooth data flow, P/R/S coordination, and real-time communication, supporting efficient and synchronized system operations across all levels.

The paper is structured into seven sections.

- Section 2 introduces the hardware architecture of the MPS assisted by MCPRS, covering its multilevel monitoring and control system based on IoT edge devices, Profibus, LAN, WAN networks, cloud, and VPN connections, along with key operational assumptions.
- Section 3 focuses on the digital twin (DT) representation of the MPS and MCPRS, including task planning using augmented reality (AR) and Synchronized Hybrid Petri Nets (SHPN) models. The formalism and virtual reality (VR) simulation of P/R/S processes and MCPRS movements are also discussed.
- Section 4 details the SCADA system, HMI synchronization, cloud and VPN integration, and the framework for real-time control.
- Section 5 is a discussion that offers insights into the approach’s effectiveness, user experience, cybersecurity, review of the literature in the field, and two other two approaches for comparison.
- Section 6 provides a review of the DT literature using PRISMA and the Systematic Literature Review (SLR).
- Section 7 presents the conclusions, summarizing key contributions and final remarks.

## 2. Hardware Architecture of MPS with MCPRS Assistance

### 2.1. Main Devices and Functionalites

**Figure 1:**
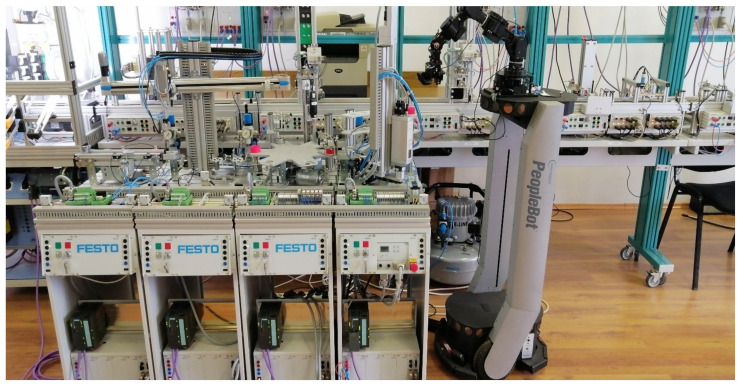
Four-WSs MPS 200 assisted by MCPRS; PeopleBot WMR equipped with Cyton 1500 RM and MVSS with Logitech camera.

**Figure 2:**
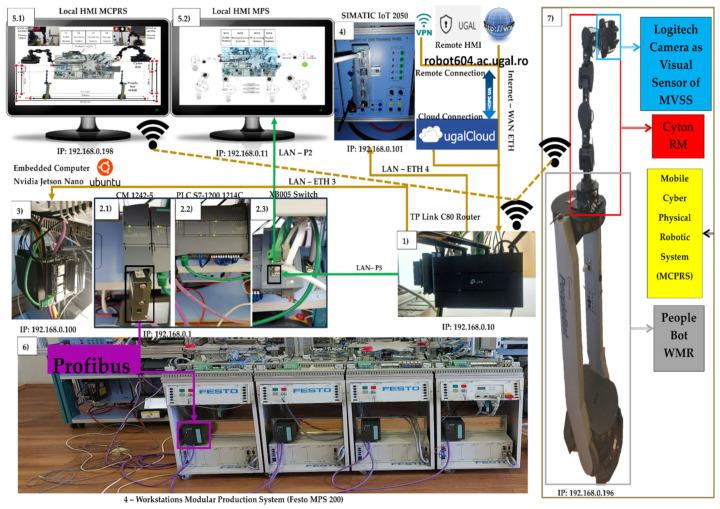
IoT edge devices, Profibus DP, LAN Profinet, LAN Ethernet, WAN Ethernet, and networking.

The modular production system shown in Figure 1, consists of four WSs that handle processes such as buffering, handling, processing, sorting/storage, reprocessing, and scrapping. These WSs are controlled by separate Siemens S7 300 PLC, connected via Profibus, to a master PLC S7-1200; each of them ensures operations in different stages, as is shown in Figure 2. This system is assisted by MCPRS which includes 2DW/1FW PeopleBot WMR, 7-DOF Cyton 1500 RM, mounted on the WMR, and MVSS with a Logitech camera mounted on the last link of the Cyton RM.

Siemens S7-1200 PLC serves as the master PLC in this system whose role is as follows:

- Coordinate and control the overall production flow across the four WSs.
- Control communication between the MPS’s WSs (buffer, handling, processing, and sorting/storage) and MCPRS.
- Control task synchronization between MPS operation and MCPRS.
- Control interfaces with remote systems over OPC-UA, WAN Ethernet, and other networks for cloud-based SCADA and HMI control.

Each MPS’s WS is controlled by a dedicated SIEMENS S7-300 PLC, which functions as a slave PLC, whose role is as follows:

- Be responsible for the operations within its respective WS.
- Communicate with the master S7-1200 PLC via Profibus, which facilitates synchronized, real-time control of individual WS’s tasks.
- Send the status, updates, and task completion to the master PLC, allowing centralized decision-making.

### 2.2. IoT Edge Devices, Profibus, Profinet, LAN Ethernet, WAN Ethernet, and Networking

In Figure 2, the devices connected to the Profibus and LANs are shown, Profinet and Ethernet, with each device having its own unique IP address. (1). WI-FI router has the role of WI-FI network management, assuring LAN Profinet, LAN Ethernet, and WAN Ethernet connectivity to the cloud via the Internet. (2.1). Communications module SIMATIC CM 1242-5, attached to S7-1214 PLC, is used for the connection of the SIMATIC S7-300 PLCs to Profibus (DP slave module). (2.2). SIEMENS PLC 1214C DC/DC/DC, as the master and HMI’s main control unit, allows all other devices to communicate with it and manages all signals (on sensors or from communication) and internal variables which are used by remote and local SCADA system. (2.3). Communication adapter XB005 Switch, attached to S7-1214 PLC is used for connecting, via LAN Profinet, the following devices to digital ports: to P2, secondary local SCADA-HMI MPS-MPS display devices; to P5, WI-FI router. (3). Embedded computer NVIDIA Jetson Nano has a Virtual Network Computing (VNC) server running on it, and VNC Viewer is enabled for remote connection. Jetson Nano has access to all the other devices and web browsers for the Node-RED interface. (4). SIEMENS IOT2050 is used as a remote SCADA System to send/receive data to/from PLC. (5.1). Local SCADA HMI MPS-MCPRS display device monitors and controls MCPRS and its interaction with MPS workstations, as well as WS4 and WS1by synchronization signals. (5.2). Local SCADA HMI MPS-MPS display device monitors and controls MPS’s WSs and their interaction with MCPRS, especially with WS4 and WS1 by synchronization signals. (6). There is a dedicated slave SIEMENS S7-300 PLCs, one for each WS.

### 2.3. Profibus, LAN Profinet, LAN Ethernet, and WAN Ethernet Communication Networks

The communication system is robust and supports real-time control and monitoring across the MPS WSs and the MCPRS. Profibus DP is a deterministic communication protocol used to connect four Siemens S7-300 PLCs at each WS to the central Siemens S7-1214 PLC, enabling high-speed data transmission and precise control. LAN Profinet is the local industrial Ethernet network used for high-speed, real-time communication between the main controller (S7-1200) and other devices, ensuring deterministic communication in industrial operations. LAN Ethernet handles general data traffic that is not time-sensitive, allowing for reliable data communication between less critical devices. WAN Ethernet provides an external internet connection, enabling the system to be monitored and controlled remotely via the VPN. OPC-UA is a standardized communication protocol used for secure and reliable communication between industrial devices. OPC-UA is particularly useful for connecting the system to cloud services and ensuring interoperability between different manufacturers’ devices.

### 2.4. Multilevel Architecture of MPS Assisted by MCPRS

**Figure 3:**
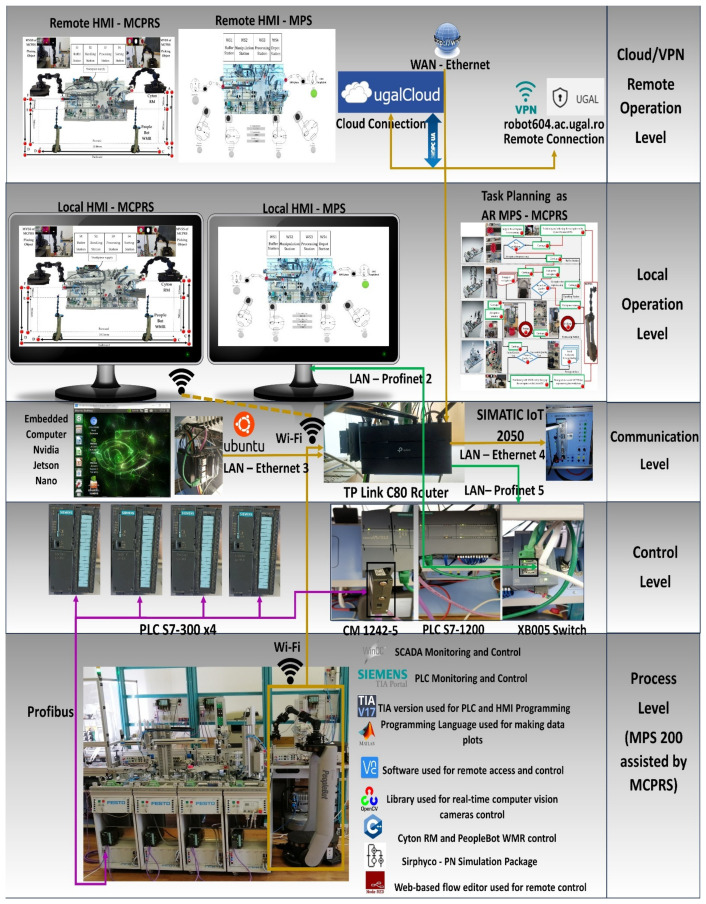
Five levels of architecture for remote or local control of MPS assisted by MCPRS.

The control structure, shown in Figure 3, is multileveled as follows.

1. Cloud/VPN remote operation level. This level includes SCADA and two HMI systems (HMI-MPS and HMI-MCPRS) for both the MPS 200 and the MCPRS. Through a secure VPN connection, users can monitor and control the entire system remotely, accessing real-time data, triggering commands, or troubleshooting any issues. Node-RED enhances this level by providing a customizable dashboard for AR-based task planning, allowing operators to visualize workflows and system performance remotely.
2. Local operation level and task flow. On-site task planning is performed using a chart flow system that coordinates and manages processes. Local SCADA/HMIs allow the user at the site to monitor and interact with the system via local interfaces, HMI-MPS and HMI-MCPRS, making it easy to adjust tasks and oversee processes in real time.
3. The communication-level and embedded computer, Jetson Nano, acts as an on-board computer for real-time data processing and vision processing for the MVSS, and controls the robotic tasks of the MCPRS. The TP-Link C80 router handles communication within the local network and connects to external networks for broader data exchange. The SIEMENS-IoT 2050 gateway facilitates communication between industrial devices (PLCs, sensors, and actuators) and the cloud, enabling seamless integration with cloud-based services and remote control.
4. Control level and Siemens S7-1214 PLC as the main control unit. This is the primary control unit for the entire system. It manages the operation of the MPS 200 and communicates with the other PLCs and devices. The CM 1242 Profibus DP adapter connects the S7-1200 PLC to the distributed S7-300 PLCs across the four workstations via the Profibus DP protocol, enabling fast and reliable control across the network. The switch manages Ethernet traffic between devices in the control network. Four Siemens S7-300 PLCs: Each workstation (buffer, handling, processing, and sorting/storage) is controlled by a dedicated Siemens S7-300 PLC, providing precise control over the respective task processes.
5. Process level, MPS 200 WSs, and MCPRS. Each of the four workstations (buffer, handling, processing, and sorting/storage) handles a specific phase in the production line, with the S7-300 PLCs coordinating the operations at each station.

**Figure 4:**
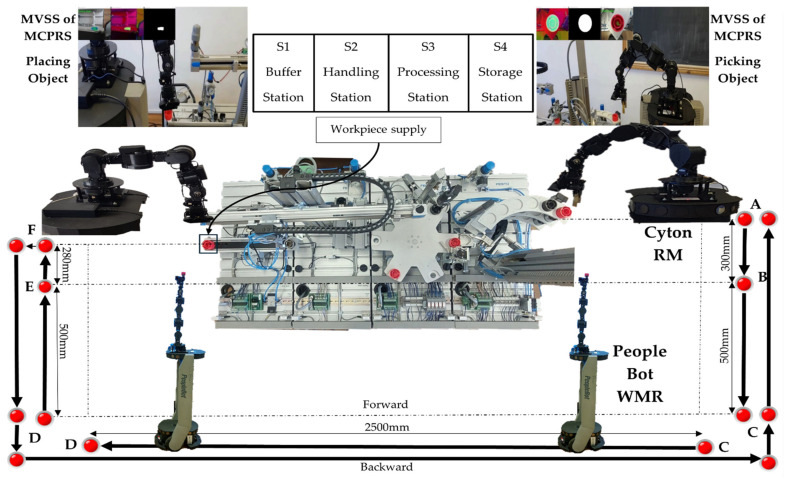
WP along MPS’s workstations, MCPRS’s movements, and WP’s picking and placing by RM and MVSS.

The control MCPRS within the modular production environment is multifaceted, integrating both discrete and continuous control systems to manage the mobility, manipulation, and visual servoing required for MCPRS tasks. The purpose is to allow the MCPRS to assist in operations across the WS1 and WS4 along the MPS 200, especially for transporting, reprocessing, and scrapping defective WPs, as is shown in Figure 4.

A single type of workpiece (WP), available in three colors (silver, red, and black), is processed, reprocessed, or scrapped, as the P/R/S technology is tailored for operations specific to this product type.

- Silver WP is assumed to pass the PQT and is stored on the middle or bottom shelf of the storage station (WS4).
- Red or black WP is assumed to fail the PQT and are placed on the top shelf of WS4, where they are retrieved by the MCPRS (as shown in Figure 4).
- Red WP is considered suitable for reprocessing after passing the SQT.
- Black WP is assumed to fail the SQT and is classified as rejected, being stored in the handling station.

To ensure seamless operation, several assumptions must be made regarding the manufacturing process on the MPS and its assistance by the MCPRS.

### 2.5. Assumptions for P/R/S Operations on MPS

**Assumption** **1.** *The MPS operates as a deterministic system governed by a single SHPN model, ensuring the production of high-quality products and enabling reprocessing for recoverable workpieces.*

**Assumption** **2.** *Only one type of WP is processed, differentiated by its color (silver, red, or black), as the system is designed for specific processing operations tailored to a single product type.*

**Assumption** **3.** *Silver WP is assumed to pass the PQT after processing and is stored on the middle or bottom shelf of the storage station (WS4).*

**Assumption** **4.** *Red or black WP is assumed to fail the PQT and is stored on the top shelf of the WS4, from where they are retrieved by the MCPRS.*

**Assumption** **5.** *Red WP is considered eligible for reprocessing after passing the SQT.*

**Assumption** **6.** *Black WP is assumed to fail the second quality test, is classified as rejected, and is stored in the handling station for scrapping.*

**Assumption** **7.** *All technological parameters, task durations, costs, and WP quantities, are known in advance.*

**Assumption** **8.** *The system operates in a pipeline mode for continuous part supply and processing, which halts temporarily when the MCPRS delivers a WP for reprocessing or scrapping.*

### 2.6. Assumptions for MCPRS-Assisted P/R/S Operations

**Assumption** **9** *The MCPRS waits for reprocessing tasks near the WS4.*

**Assumption** **10.** *MCPRS transports WP from the WS4 to the WS1 along a predefined route at a constant speed.*

**Assumption** **11.** *After delivering the WP to the buffer station, the MCPRS returns via the same route to the storage station.*

**Assumption** **12.** *The positioning of the WP during pickup and drop-off is managed using the 7-DOF Cyton RM and MVSS for precise handling.*

**Assumption** **13.** *The MCPRS operates in an obstacle-free environment to ensure seamless movement.*

## 3. The Virtual World as a Digital Counterpart of P/R/S Technology on MPS Assisted by MCPRS

### 3.1. AR and VR as Components of the Virtual World

**Figure 5:**
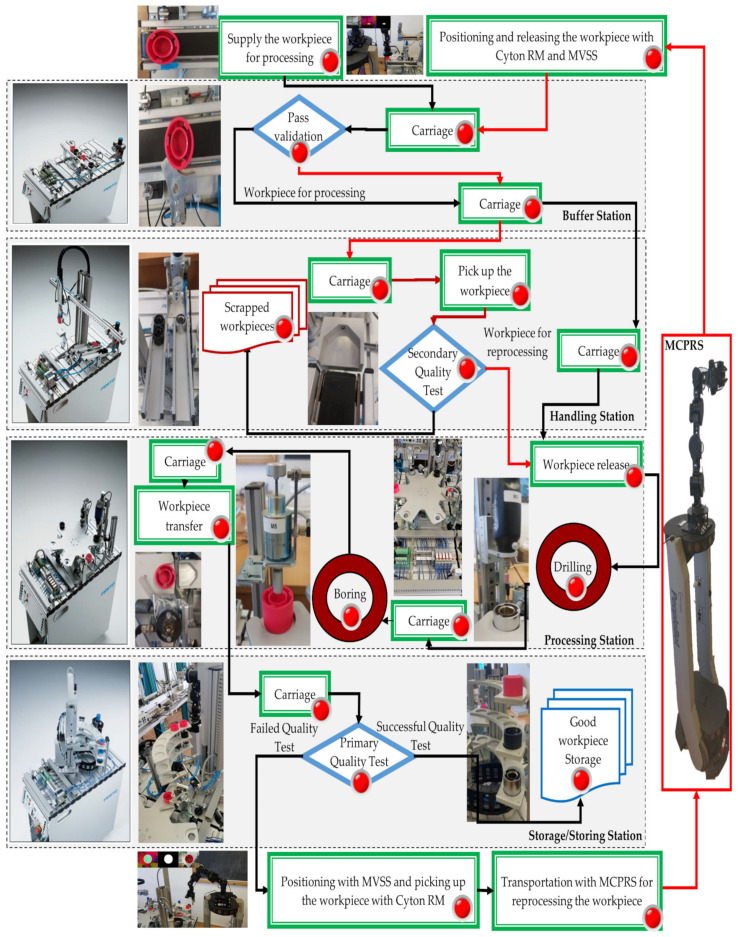
Node-RED, P/R/S task planning augmented reality.

The virtual world, as the counterpart of the digital twin of P/R/S technology on MPS assisted by MCPRS, has two components:

- AR with Node-RED, which is AR associated with task planning by using Node-RED functions (Figure 5) [28].
- VR with SHPN to model P/R/S workflow by using SHPN simulation and the Sirphyco package [29].

Node-RED allows for the creation of AR dashboards and task management systems. Using this tool, users can design workflows, visualize task execution in real time, and receive alerts or updates about the system’s performance. It simplifies the programming of automation flows and integrates well with the SCADA/HMI systems. Task planning is conceived as an AR implemented as a flow of Node-RED functions that are transposed in two HMIs, one for the operations performed on MPS, and another for MCPRS assistance. The tasks in the organization chart are executed synchronously using PLC signals. The execution of a task corresponds to a monitoring signal which in the organizational chart is signaled by lighting the spotlight. Task planning is enhanced with AR technology, allowing users to interact with the production system visually. Through Node-RED functions, the user can visualize real-time processes and plan or modify tasks by interacting with virtual workstations and robotic systems. The user can observe how the MCPRS moves, picks, and places WP, or how each workstation handles its respective processing task. The user can also simulate workflow changes to predict performance.

The Sirphyco package is used to simulate the SHPN model, enabling the system to model workflow, resource dependencies, and the synchronization between discrete and continuous tasks. TPNs manage the operations and task sequences at the MPS 200 workstations. CPNs control the continuous and smooth movements of the MCPRS, optimizing its trajectory, speed, and interaction with the production system.

### 3.2. Workflow, Task Planning, Synchronization, and SHPN Structure

The WP starts at WS1 (Buffer) and moves successively through WS2 (Handling), WS3 (Processing), and WS4 (Storage/Sorting). At each station, the respective task is controlled by a TPN model that dictates the timing and sequencing of operations.

PQT at WS4: If the WP passes quality inspection, it is sorted and stored. If it fails, it must be reprocessed or scrapped. Reprocessing or Scrapping via MCPRS: The MCPRS is activated if the WP requires reprocessing or scrapping. The MVSS mounted on the Cyton RM inspects the WP to determine its condition. Using CPN models, the MCPRS moves to WS4 and picks up the defective WP. It then transports the WP back to WS1 for reprocessing, or it moves to a scrap part.

SQT at WS2: If the WP passes the SQT, it goes on to WS3 for reprocessing (drilling and boring) and to WS4 for storage, otherwise it is stored on the slide as a scrap part.

Synchronization between MPS and MCPRS: The SHPN simulation ensures that both MPS and MCPRS operate in coordination, with tasks synchronized across the system. This synchronization prevents collisions, manages shared resources, and optimizes task completion time.

**Figure 6:**
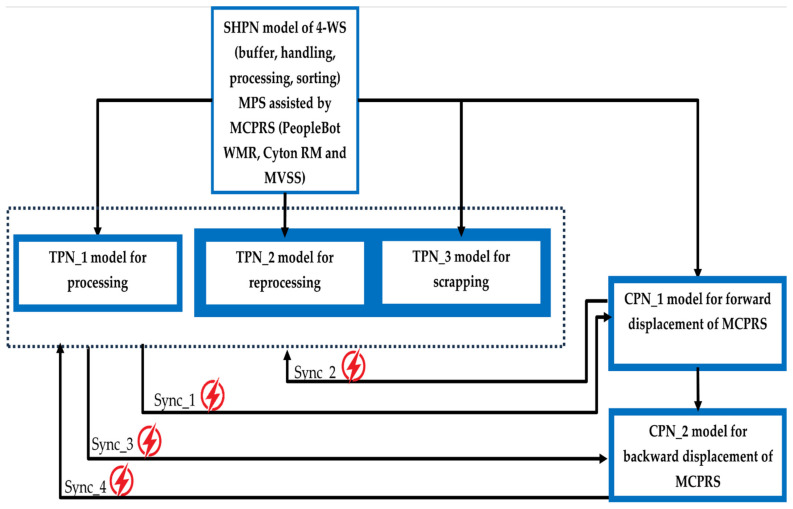
Structure of SHPN model: TPN_1, TPN_2, and TPN_3 for P/R/S on MPS, and CPN_1 and CPN_2 for MCPRS movements.

The hybrid nature of the P/R/S technology operating on the MPS with MCPRS assistance is characterized by the MCPRS’s movement between the storage station (last WS) and the buffer station (first WS), in both forward and backward directions, as illustrated in Figure 4. The task planning integrates the discrete operations of the MPS with the continuous motion of the MCPRS, forming a SHPN model, as demonstrated in [1,2,3]. The SHPN model enables synchronization with external events, such as signals from sensors and the mobile visual servoing system (MVSS), ensuring real-time coordination. The structure, depicted in Figure 6, combines the following:

- Discrete elements for modeling MPS operations: TPN_1 for processing, TPN_2 for reprocessing, and TPN_3 for scrapping.
- Continuous elements for MCPRS movements: CPN_1 for forward motion and CPN_2 for backward motion.

**Figure 7:**
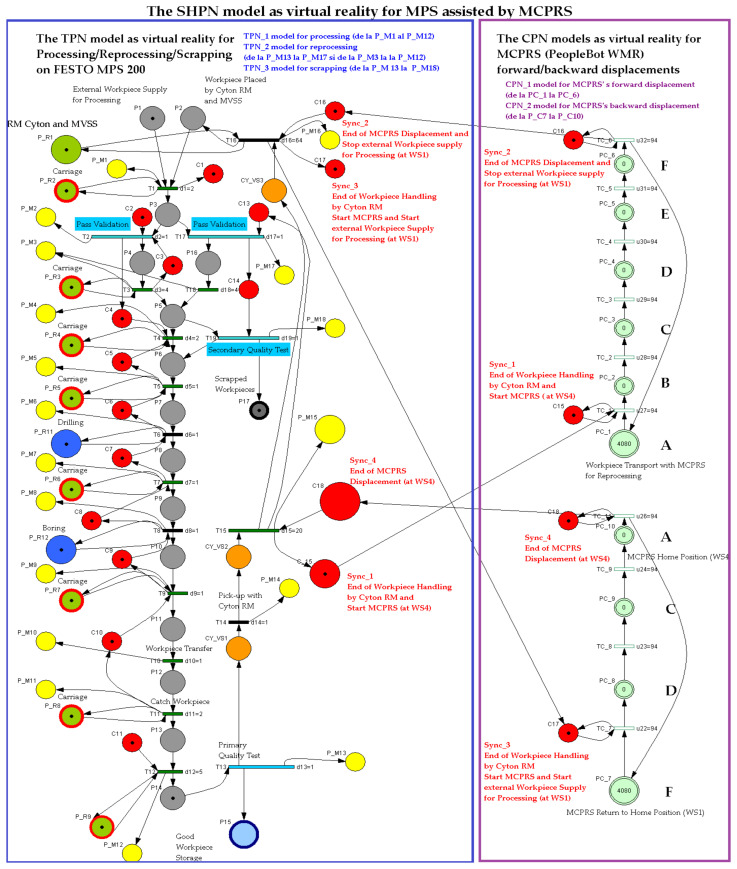
SHPN model (TPN_1, TPN_2, TPN_3, CPN_1, and CPN_2) of P/R/S operations on MPS assisted by MCPRS.

With the inclusion of external events from sensors, which synchronize the MPS with MCPRS subsystems, the final model is classified as SHPN, as shown in Figure 7.

### 3.3. SHPN Together with TPN and CPN Models, Formalism, and Simulation

The SHPN model based on task planning shown in Figure 5 and structure shown in Figure 6, is an oriented graph described with SHPN formalism [5,6].

The SHPN model describes both discrete and continuous dynamics corresponding to the P/R/S operations on MPS and MCPRS assistance. The discrete model corresponds to the P/R/S, while the continuous model corresponds to the MCPRS displacement for picking and placing the WP for reprocessing or scrapping. Thus, the model becomes a hybrid one, as shown in Figure 7.

The system’s behavior is modeled with SHPN and simulated with the Sirphyco package. This is particularly effective for validating workflows, task synchronization, and dynamic system behavior. TPN_1, TPN_2, and TPN_3 are used to control the operations of the MPS’s WSs, ensuring synchronization between different process stages. CPN_1 and CPN_2 govern the continuous movements and actions of the MCPRS, optimizing its path and task execution in relation to the WSs.

In the SHPN model, shown in Figure 7, the states in red are control states associated with the control functions of the decision-making, being states that trigger a transition when they receive a token. The states in brown or gray correspond to the pick-and-place actions and obtain the token at the end of the transition. Yellow states correspond to monitoring actions and obtain the token after buffering, handling, drilling, boring, sorting, or transport transition has been completed. There are also four states in red, which are synchronization signals that receive a token when one of MCPRS actions has been completed, conditioning the start of another. The simulation of a STPN model is performed in the Sirphyco package [2,29].

SHPN model, from Figure 7, associated with MPS assisted by MCPRS is a triplet,

(1) $$\mathrm{SHPN}=\begin{array}{ccc} \langle \mathrm{THPN}\left(\mathrm{TPN}\_1,\mathrm{TPN}\_2,\mathrm{TPN}\_3,\mathrm{CPN}\_1,\mathrm{CPN}\_2\right), & E_{d}, & \mathrm{Sync}\rangle \end{array},$$

where THPN is a septuplet,

(2) $$\mathrm{THPN}\left(\mathrm{TPN}\_1,\mathrm{TPN}\_2,\mathrm{TPN}\_3,\mathrm{CPN}\_1,\mathrm{CPN}\_2\right)=\langle P,\;T,\;\mathrm{Pre},\mathrm{Post},{\;m}_{o},\;h,\;\mathrm{tempo}\rangle$$

TPN_1 is the discrete model for processing.

TPN_2 is the discrete model for reprocessing.

TPN_3 is the discrete model for scrapping.

CPN_1 is the continuous model for MCPRS forward displacement.

CPN_2 is the continuous model for MCPRS backward displacement.

$E_{d}$ is a set of external events, which are signals from sensors.

Sync maps the elements for the set of discrete transitions in the set of external events. External events are signals from sensors, used for synchronization,

(3) $$\mathrm{Sync}:\;T\to E_{d}\;$$

P is a set of finite places,

(4) $$P=P^{D}\cup P^{C},$$

where is the set of the discrete transitions for the WP2 repair tasks,

(5) $$P^{D}=\left\{P_{P\_R_{1}}\right\}\cup {\left\{P_{P_{i}}\right\}}_{i=\bar{1,17}}\cup {\left\{P_{C_{i}}\right\}}_{i=\bar{1,18}}\cup {\left\{P_{\mathrm{CY}\_{\mathrm{VS}}_{i}}\right\}}_{i=\bar{1,3}}\cup {\left\{P_{P\_M_{i}}\right\}}_{i=\bar{1,18}}$$

with $\left\{P_{P\_R_{1}}\right\}$—the state that receives the token when a work piece is deposited on WS1; ${\left\{P_{P_{i}}\right\}}_{i=\bar{1,17}}$ (gray)—the set of the discrete states corresponding to the P/R/S operations on the MPS, (buffering, carriage, handling, catching, drilling, boring, sorting, and storage), except that $\left\{P_{P_{15}}\right\}$ (light blue)— the state that receives a token when a work piece is deposited on WS4, as good; $\left\{P_{P17}\right\}$ (dark gray)— the state that receives a token when a work piece is submitted to WS2, as being scrapped; ${\left\{P_{C_{i}}\right\}}_{i=\bar{1,18}}$ (red)—the set of discrete states associated with the control functions related to some decision-making actions of the control system, except the last four, which are synchronization signals between MPS and MCPRS.

${\left\{P_{\mathrm{CY}\_{\mathrm{VS}}_{i}}\right\}}_{i=\bar{1,3}}$ (orange)—the discrete state that defines the Cyton RM and MVSS of MCPRS actions picking, handling, and placing of the WP for reprocessing or scrapping); ${\left\{P_{P\_M_{i}}\right\}}_{i=\bar{1,18}}$ (yellow)—the set of monitoring states associated with the monitoring function of the successive P/R/S actions; ${\left\{P_{P\_R_{i}}\right\}}_{i=\bar{1,19}}$ (light green)—the state set corresponding to transfers between different locations of P/R/S operations.

(6) $$P^{C}={\left\{P_{{P\_R}_{k}}\right\}}_{k=\bar{1,9}}\cup {\left\{P_{P\_C_{k}}\right\}}_{k=\bar{1,6}}\cup {\left\{P_{P\_C_{k}}\right\}}_{k=\bar{7,10}},$$

${\left\{P_{{P\_R}_{k}}\right\}}_{k=\bar{1,9}}$ (light green) is the set of continuous states associated with transporting the workpiece along the MPS for P/R/S operations, except $\left\{P_{P\_R_{11}},P_{P\_R_{12}}\right\}$ (dark blue) which corresponds to processing, drilling, and boring operations;$\;{\left\{P_{P\_C_{k}}\right\}}_{k=\bar{1,6}}$ is the continuous place associated with forward and ${\left\{P_{P\_C_{k}}\right\}}_{k=\bar{7,10}}$ with backward displacement of MCPRS.

T is the set of finite transitions

(7) $$T=T^{D}\cup T^{C},$$

where

(8) $$T^{D}={\left\{T_{d_{i}}\right\}}_{i=\bar{1,12}}\cup \left\{T_{r_{14,15,16}}\right\}\cup \left\{T_{{\mathrm{PQT}}_{13}},T_{{\mathrm{SQT}}_{19}}\right\}\cup \left\{T_{{\mathrm{PV}}_{17}},T_{18}\right\},$$

${\left\{T_{d_{i}}\right\}}_{i=\bar{1,12}}$ is the set of the discrete transitions for the execution of P/R operations, exception $\left\{T_{d_{2}}\right\}$ which is the transition corresponding to pass validation, $\left\{T_{{\mathrm{PV}}_{2}}\right\}$, of the WP for processing; $\left\{T_{r_{14,15,16}}\right\}$ is the set of the discrete transitions of MCPRS actions (piking, displacement and placing of the WP for reprocessing or scrapping); $\left\{T_{{\mathrm{PQT}}_{13}},T_{{\mathrm{SQT}}_{19}}\right\}$ is the set of the discrete transitions for PQT and SQT; and $\left\{T_{{\mathrm{PV}}_{17}},T_{18}\right\}$ is the set of the discrete transitions for pass validation and transporting on the conveyor belt of the WP for reprocessing or scrapping.

(9) $$T^{C}={\left\{T_{c_{k}}\right\}}_{k=\bar{1,6}}\cup {\left\{T_{c_{k}}\right\}}_{k=\bar{7,11}}$$

where ${\left\{T_{c_{k}}\right\}}_{k=\bar{1,6}}$ is the set of the continuous transitions for forward (from WS4 to WS1) and ${\left\{T_{c_{k}}\right\}}_{k=\bar{7,11}}$ for backward (from WS1 to WS4) MCPRS’s displacements for reprocessing or scrapping. The sets of the places and of the transitions are disjoint P∩T=O.

Pre is the input incidence function, $\mathrm{Pre}:P\times T\to Q_{+}$.

Post is the output incidence function, $\mathrm{Post}:P\times T\to Q_{+}$.

m_o_ is the initial marking, $m_{\;o}:P\to R_{+}$.

h is a hybrid function,

(10) $$h:P\cup T\to \left\{D,\;C\right\},\;h:P^{D}\cup T^{D}\to \left\{D\right\};\;h:P^{C}\cup T^{C}\to \left\{C\right\}.$$

tempo is a function that defines the time durations associated with the transitions, from (8) and (9). $\mathrm{tempo}:T\to Q_{+}\cup \left\{0\right\}$

To synchronize the P/R/S operations from the input of WS1 and from the output of WS4 with MCPRS, four control signals issued by the master PLC on Profibus are required. In the SHPN model and formalism, the appearance of these signals is highlighted by a set of events, demonstrated as follows,

(11) $$E_{d}={\left\{{\mathrm{Edd}}^{i}\right\}}_{i=\bar{1,4}}\cup \left\{e\right\}$$

where ${\mathrm{Edd}}^{1}=\mathrm{Sync}\left(P_{C_{1}}\right)$, ${\mathrm{Edd}}^{2}=\mathrm{Sync}\left(P_{C_{6}}\right)$, ${\mathrm{Edd}}^{3}=\mathrm{Sync}\left(P_{C_{7}}\right)$, and ${\mathrm{Edd}}^{4}=\mathrm{Sync}\left(P_{C_{10}}\right)$, as is shown in Figure 6.

$\left\{e\right\}$ represents the neutral event that synchronizes all the discrete transitions in (8) and that are neutral in terms of synchronization with other subprocesses.

${\left\{{\mathrm{Edd}}^{i}\right\}}_{i=\bar{1,4}}\cup \left\{e\right\}$ are the signals injected by changing the states, in the controlled sub-processes, as follows:

(12) $$Sync\_1:T_{15}\to \left\{{\mathrm{Edd}}^{1}\right\},$$

(13) $$Sync\_2:T_{16}\to \left\{{\mathrm{Ed}}^{2}\right\},$$

(14) $$Sync\_3:T_{16}\to \left\{Ed^{3}\right\},$$

(15) $$Sync\_4:T_{15}\to \left\{{\mathrm{Ed}}^{4}\right\},$$

${\mathrm{Edd}}^{1}$ synchronization signal for **Sync_1:** “**End of WP Handling by Cyton RM and Start Forward Displacement of MCPRS (at WS4)**”.

${\mathrm{Edd}}^{2}$ synchronization signal for **Sync_2:** “**End of Forward Displacement of MCPRS and Stop External WP Supply for Processing (at WS1**)”.

${\mathrm{Edd}}^{3}$ synchronization signal for **Sync_3:** “**End of WP Handling by Cyton RM Start Backward Displacement of MCPRS and Start External WP Supply for Processing (at WS1)**”.

${\mathrm{Edd}}^{4}$ synchronization signal for **Sync_4:** “**End of Backward Displacement of MCPRS and Wait a WP for Reprocessing or Scrapping (at WS4)**”.

Each monitoring place in (5) monitors a certain transition in the set (8) as follows: P_M1-monitors T1 (on WS1-carry); P_M2-monitors T2 (pass validation on WS1-WP for processing); P_M3-monitors T3 (on WS1, carry and transfer WS1 to WS2); P_M4-monitors T4 (onWS2, handling WP); P_M5-monitors T5 (transfer to WS3-carry); P_PM6-monitors T6 (on WS3-drilling); P_M7-monitors T7 (on WS3-carry); P_M8-monitors T8 (on WS3-boring); P_M9-monitors T9 (on WS3-carry); P-M10-monitors T10 (transfer to WS4); P-M11-monitors T11 (catch WP on WS4); P-M12-monitors T12 (on WS4-WP handling and storage); P_M13-monitors T13 (on WS4-PQT of WP); P_M14-monitors (on WS4 pick-up WP with Cyton RM); P_M15-monitors T15 (on MCPRS-${\mathrm{Edd}}^{1}$; P_M16-monitors T16 (On MCPRS-${\mathrm{Edd}}^{2}$ and ${\mathrm{Edd}}^{3}$; P_M17-monitors T17 (pass validation on WS1-WP for reprocessing or scrapping); and P_M18-monitors T18 (on WS2-SQT of WP).

**Figure 8:**
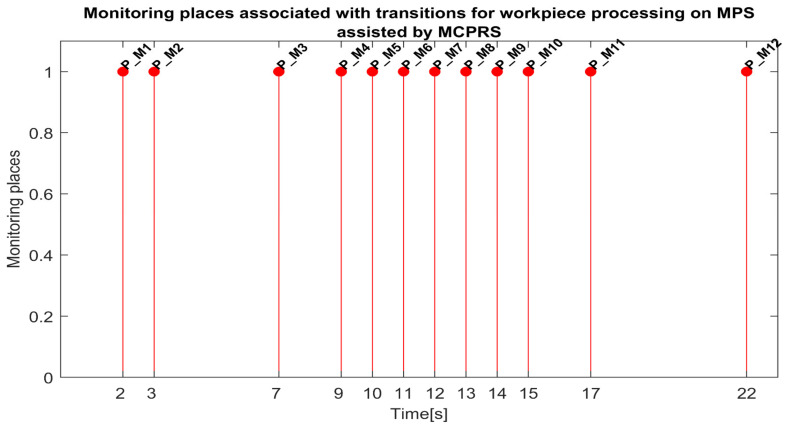
Sirphyco simulation of the TPN_1 model for processing on MPS.

The simulation in Sirphyco of the monitoring signals corresponding to the transitions of a WP for processing, ${\left\{P_{M_{i}}\right\}}_{i=\bar{1,12}}$, is shown in Figure 8 [29,30].

**Figure 9:**
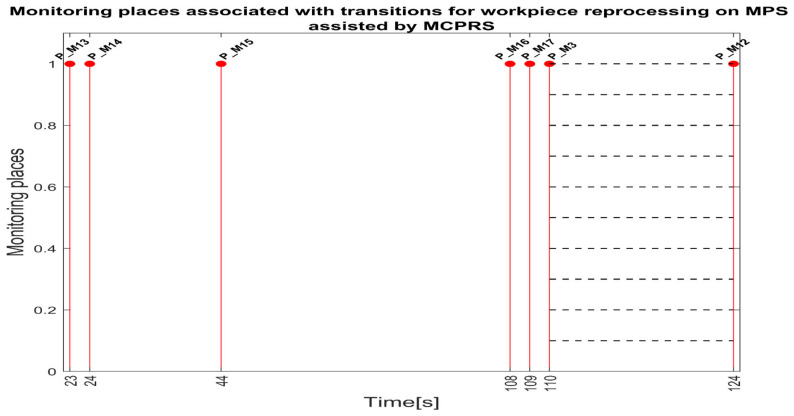
Sirphyco simulation of the TPN_2 model for reprocessing on MPS.

The simulation in Sirphyco of the monitoring signals corresponding to the transitions of WP for reprocessing, from pick (at WS4), placement (at WS1) with Cyton RM and MVSS (TPN_2), and SQT pass and processing on TPN_1, ${\left\{P_{M_{i}}\right\}}_{i=\bar{13,17}\cup i=\bar{3,12}}$, is shown in Figure 9 [29,30].

**Figure 10:**
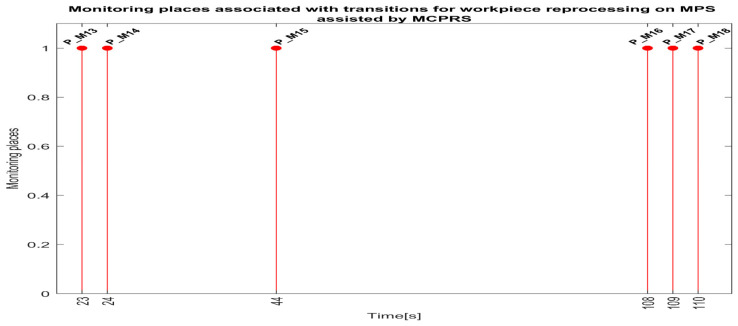
Sirphyco simulation of the TPN_3 model for scrapping on MPS.

The simulation in Sirphyco of the monitoring signals corresponding to the transitions of a WP for scrapping, from pick (from WS4), placement (at WS1) with Cyton RM and MVSS (TPN_2), and SQT fails, ${\left\{P_{M_{i}}\right\}}_{i=\bar{13,18}}$, is shown in Figure 10 [29,30].

**Figure 11:**
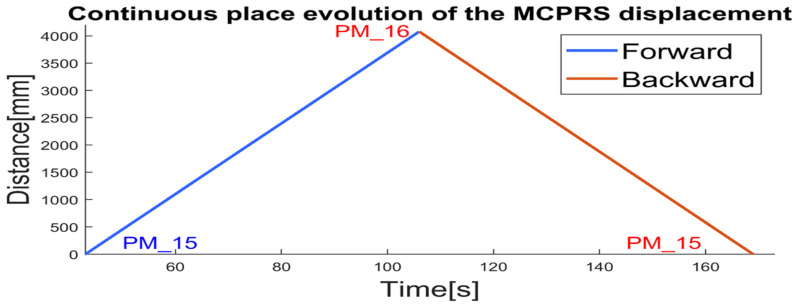
Sirphyco simulation of CPN_1 and CPN_2 models for MCPRS forward and backward displacements.

The continuous evolution of the MCPRS’s forward, $P_{c_{1}}$, and backward, $P_{c_{7}}$, displacements is depicted in Figure 11 [29,30]. Using the CPN_1 and CPN_2 models simulated in the Sirphyco package, Figure 11 illustrates the distances traveled by the MCPRS as it transports a workpiece from WS4 to WS1 for reprocessing or scrapping and then returns to WS4. In these CPN models, the remaining distance for the MCPRS is modeled as a continuous variable that changes over time at a constant speed of 94 mm/s. The MCPRS executes the following sequences at a uniform speed:

- Forward Displacement (Blue): Moving from WS4 $\left(P_{c\_1}\equiv \left(A\right)\right)$ to WS1$\left(P_{c\_6}\equiv \left(F\right)\right)$;
- Backward Displacement (Red): Returning from WS1$\left(P_{c\_7}\equiv \left(F\right)\right)$ to WS4, $\left(P_{c\_10}\equiv \left(A\right)\right)$.

### 3.4. Virtual Digital Counterpart of the MCPRS

MobileSim is used for the trajectory simulation of the MCPRS to test and validate navigation paths within the MPS environment. This allows for realistic and error-free path planning. A virtual map of the MPS layout, including all four workstations, is created in MobileSim [31]. This allows MCPRS’s virtual counterpart to identify obstacles, paths, and spatial constraints. By simulating different paths, MobileSim optimizes the trajectory for minimum travel time and collision avoidance, aligning with the tasks required at each workstation. MobileSim uses discrete-time trajectory tracking to simulate the MCPRS movement, applying parameters that mirror those in the real-world PeopleBot WMR. This enables realistic simulations of acceleration, deceleration, and turning points. The discrete-time trajectory tracking sliding mode control (DT-TTSMC) strategy used in MobileSim accounts for dynamic variations in the virtual environment, such as simulated obstacles or path deviations. This makes the trajectory more stable and adaptable, which is essential for path tracking on uneven or cluttered surfaces in the MPS. Within MobileSim, CPN is used to synchronize MCPRS’s movement with virtual MPS operations, ensuring smooth task progression and timing alignment. The MCPRS waits for workpieces designated for reprocessing near the storage station (WS4). It picks up the workpiece from WS4 and transports it to the buffer station (WS1) for either reprocessing or scrapping, following the route shown in Figure 4 at a constant speed. After delivering the workpiece to WS1, the MCPRS returns along the same route to WS4. The precise positioning of the end effector during pickup and drop-off is achieved using the Cyton RM and the MVSS. The MCPRS operates in a clear, obstacle-free environment, with this process focusing on the mobility provided by the PeopleBot WMR.

**Figure 12:**
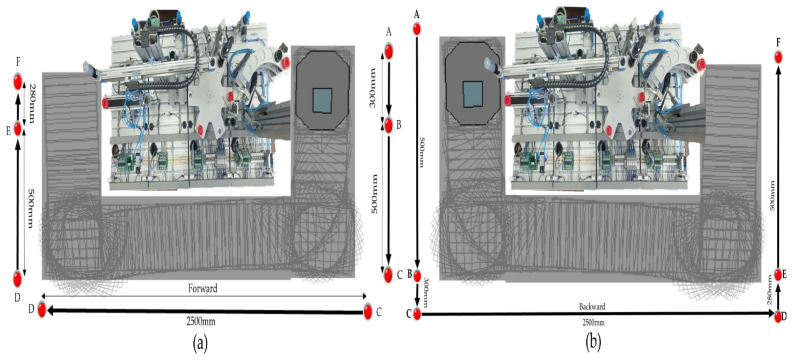
MobileSim (**a**) forward and (**b**) backward trajectories of MCPRS around MPS.

For integration with the MPS workflow, trajectory, and task synchronization, CPN1 and CPN2, simulated in Sirphyco (Figure 7 and Figure 11), are used to align the MCPRS’s movement with the virtual operations of the MPS. Virtual synchronization mirrors the tasks of transporting WP from WS4 (storage) to WS1 (buffer) for reprocessing or scrapping. End-effector control simulates precise pick-and-place tasks using Cyton RM and MVSS. Visual and positional feedback in the virtual environment ensures alignment with physical processes. The DT-TTSMC control strategy is implemented in Visual C++, VC 12.0 [32]. ARIA (Advanced Robotic Interface for Applications) functions are used for MCPRS control [33]. If the PeopleBot WMR is undetected, MobileSim automatically initiates, ensuring uninterrupted testing. The virtual forward and backward closed-loop MCPRS trajectories obtained in MobileSim to transport the WP for reprocessing or scrapping are shown in Figure 12.

## 4. Real World of P/R/S on MPS Assisted by MCPRS

### 4.1. SCADA Monitoring Signals and Syncronization

**Figure 13:**
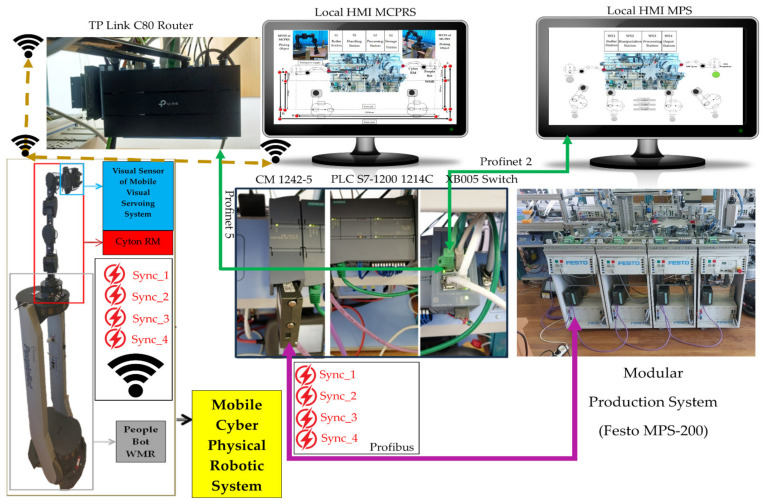
Communication block set between MPS assisted by MCPRS and local PCs. HMI-MCPRS. and HMI-MPS.

**Figure 14:**
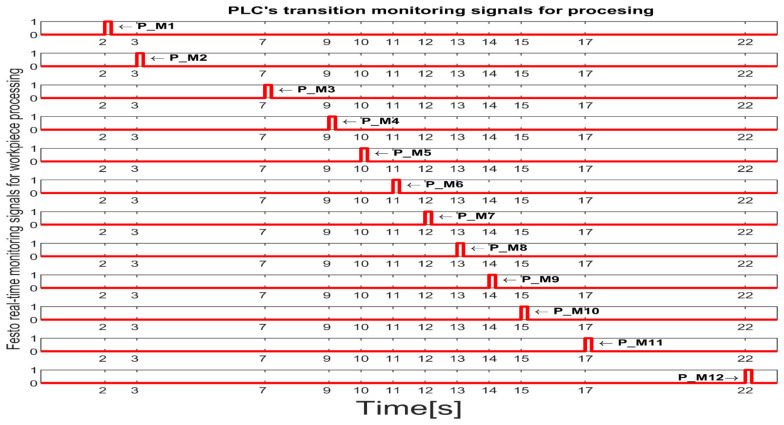
Monitoring signals from master PLC for WP’s processing.

**Figure 15:**
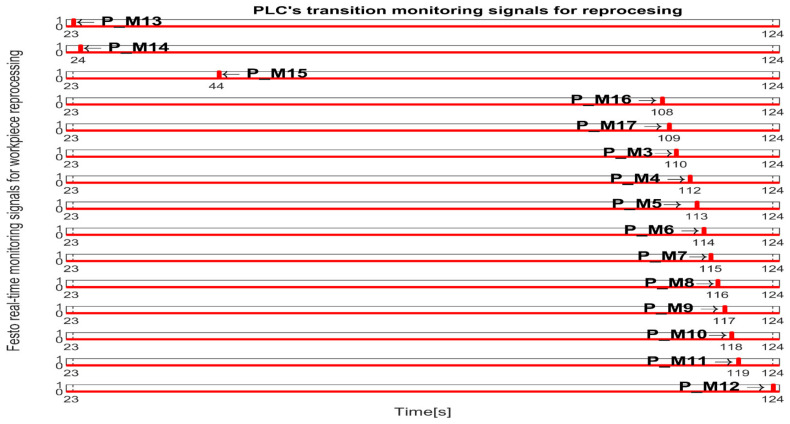
Monitoring signals from master PLC for WP’s reprocessing.

**Figure 16:**
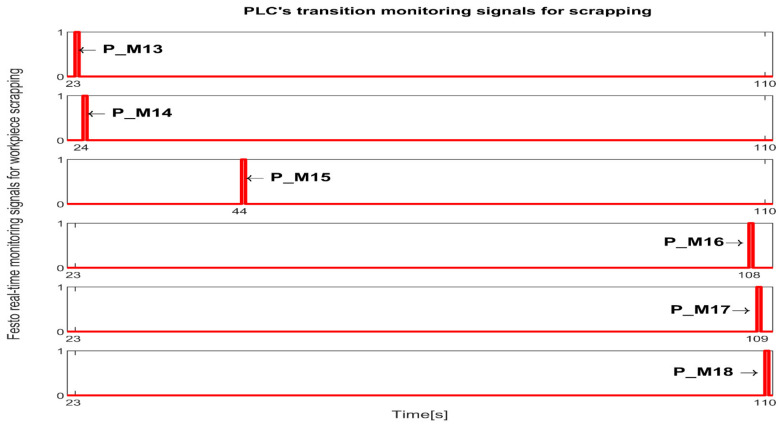
Monitoring signals from master PLC for WP’s scrapping.

The SCADA system in the cloud/VPN-based remote control of P/R/S integrates data acquisition, communication, presentation, and remote-control functionalities to ensure synchronized operations between the MPS and MCPRS. The SCADA system and HMI platforms, HMI-MPS and HMI-MPCRS, were implemented in the cloud, as shown in Figure 3, and in local Figure 2 and Figure 13, the following main functions are shown being integrated for the real-time monitoring, controlling, and visualizing of P/R/S technology [34,35]:

(1) Data acquisition, to monitor and control all I/O field sensors in the MPS and MCPRS hardware architecture. It includes the acquisition of the sensor readings from MPS WSs, WS1 to WS4, and positioning and quality signals for MCPRS tasks such as pick-and-place and transport.
(2) Data communication provides seamless interaction between devices and sensors over industrial communication protocols. Profibus DP, for MPS, utilizes the SIEMENS CM 1242-5 adapter to connect the S7-1214 master PLC with the S7-300 slave PLCs in MPS, and enables cyclic communication for transferring process data between WSs. Wireless TCP/IP for MCPRS to interfaces subsystems like PeopleBot, Cyton RM, and MVSS with SCADA via Ethernet for real-time task execution.
(3) Data presentations display real-time operational states, sensor readings, and transition conditions through dashboards on HMI-MPS and HMI-MCPRS. Visual tools include transition state visualizations for the SHPN model, graphical timelines of process and transition events, as shown in Figure 14, Figure 15 and Figure 16, and alerts for anomalies or out-of-range sensor readings.
(4) Remote and local control: SCADA transmits validated control commands to field devices for process adjustments and executes synchronization tasks for P/R/S operations, such as processing operations, transporting WPs from WS4 to WS1 for reprocessing or scrapping, activating PeopleBot DT-TTSMC control for precise displacement, and coordinating Cyton RM and MVSS actions for end-effector tasks.

Synchronization between MPS and MCPRS is essential for ensuring the timely execution of operations. The SHPN model represents states and transitions in MPS and MCPRS processes. SCADA links SHPN transitions with physical system signals, ensuring alignment of workpiece movement across workstations and timely triggering MCPRS subsystems for picking up, transport and placement. The real-time application of SCADA control signals validates SHPN transitions for workpiece picking at WS4, transport and placement at WS1, for reprocessing and scrapping [8,9]. Signals are conditioned by the SHPN model (TPN_1, TPN_2, TPN_3, CPN_1, and CPN_2), as shown in Figure 6, Figure 8, Figure 9 and Figure 10, and appear in real-time monitoring, as shown in Figure 14, Figure 15 and Figure 16. The coordination of MPS and MCPRS minimizes process cycle times. Real-time feedback through SCADA aids with identifying bottlenecks or delays. The HMI-MPS dashboard focuses on monitoring MPS’s WS activities, including P/R/S states, quality assurance, PQT, SQT, and data logs for sensor signals and transitions. The HMI-MCPRS dashboard tracks MCPRS subsystems PeopleBot WMR movement and orientation, Cyton RM end-effector positioning, and MVSS image-based feedback for precise alignment. Both cloud-based (Figure 3) and local (Figure 2 and Figure 13) implementations allow users to switch seamlessly between remote and local control, ensuring redundant access for critical processes. Figure 14, Figure 15 and Figure 16 illustrate the real-time signals recorded during P/R/S operations. A comparison with simulation results, as shown in Figure 8, Figure 9 and Figure 10, shows consistent time intervals for transitions, validating system performance. We compared the transition monitoring signals in Figure 8, Figure 9 and Figure 10 that were obtained from the simulation via Sirphyco of the TPN_1, TPN_2 and TPN_3 models, in Figure 7, with the signals obtained in real time through the SCADA system, in Figure 14, Figure 15 and Figure 16, respectively. It can be observed that they appear at approximately the same time intervals, marking the end of the duration of the transitions corresponding to the P/R/S operations.

The system architecture resembles IoT with embedded sensors and controllers, integrating computational and physical elements to ensure synchronized operation and real-time adaptability. The combination of computational and physical elements makes MCPRS analogous to IoT systems. This robust SCADA-based monitoring and synchronization framework ensures the seamless operation of the MPS and MCPRS for P/R/S technology, enhancing the overall system’s efficiency and flexibility.

### 4.2. Real-Time Control of MCPRS

**Figure 17:**
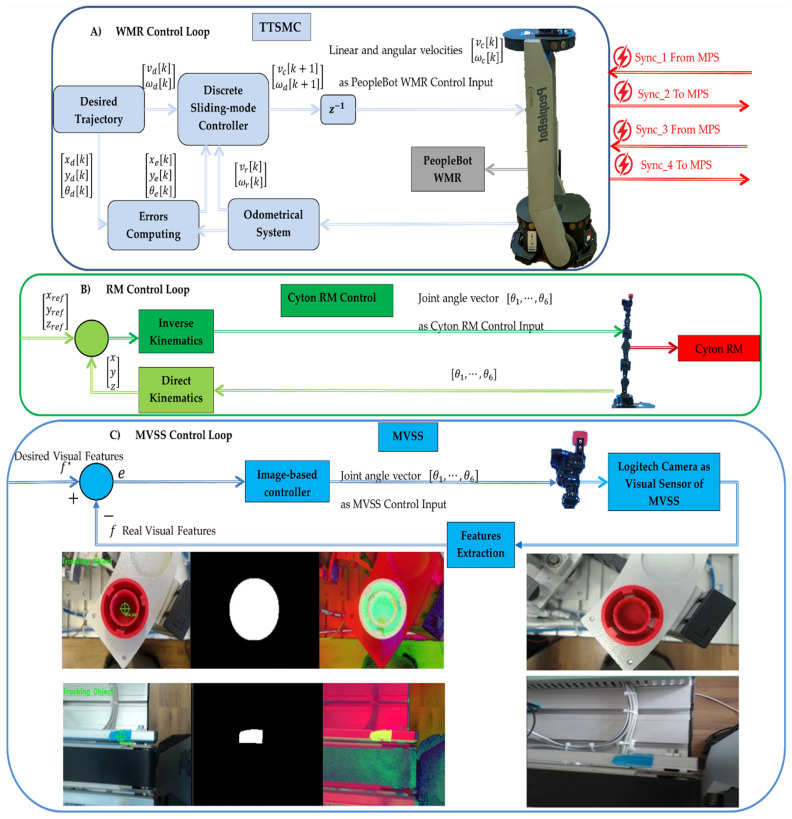
MCPRS control loops. (**A**) PeopleBot WMR control loop. (**B**) Cyton RM control loop. (**C**) MVSS control loop.

The control system for the MCPRS utilizes three hierarchical control loops that ensure the effective functioning of its key components: PeopleBot WMR, Cyton RM, and MVSS, as shown in Figure 17. These loops coordinate tasks like transportation, manipulation, and visual guidance in real time through a combination of local and remote interactions with PC-HMI dashboards and the SCADA system.

(A) The first control loop (PeopleBot WMR control) controls the movement of the PeopleBot WMR for forward and backward motion between WS4 and WS1. The control method is DT-TTSMC. This ensures precise trajectory tracking under dynamic conditions and integrates real-time data from the robot’s odometer system and onboard. Communication uses the ARIA Mobile Robots Library for command execution, which communicates wirelessly with the remote or local PC-HMI via the SCADA system [36]. Position and feedback data are transmitted from the embedded microcontroller via Wi-Fi to the SCADA server, which computes and sends control commands back to the WMR.
(B) The second control loop (Cyton RM command synchronization). Handles the synchronization of commands between the Siemens S7-1200 PLC, and the Cyton RM. The control method is Modbus TCP communication which uses standard industrial protocols for real-time coordination between PLCs and the Cyton RM. This control loop ensures smooth task execution for robotic arm manipulation. Commands are wirelessly transmitted between the PC-HMI-MCPRS and the Cyton RM through an Ethernet adapter using a specific TCP/IP protocol.
(C) The third control loop (MVSS control for end-effector accuracy) controls the movement of the MVSS to enhance the accuracy of pick-and-place tasks performed by Cyton RM’s end-effector. The control method is an image moments method that processes real-time image data to guide the end-effector in precise positioning. This control uses wireless communication to interface between the PC-HMI-MCPRS and MVSS. The communication is based on real-time updates sent wirelessly to adjust the positioning dynamically.

The role of SCADA and HM-MCPRS in coordination consists of the PC-HMI-MCPRS functioning as a SCADA server to synchronize data from all three control loops, to coordinate between the PeopleBot WMR, Cyton RM, and MVSS and transmit commands wirelessly to the MPS’s Siemens S7-300 PLCs for integration with WSs activities. SCADA extends monitoring and control to remote locations via cloud-based VPN connections, facilitating real-time decision-making and supervisory tasks.

The cyber–physical integration of MCPRS consists of a combination between physical elements, WMR, RM, MVSS, and computational processes, SCADA, HMIs, and wireless communication. IoT-like features share basic IoT architecture, with a seamless interface between computational and physical layers, and leverage real-time feedback from embedded systems for adaptive control.

Advantages of this real-time control architecture include the following:

Precision by DT-TTSMC ensures accurate trajectory tracking and smooth operation.

Flexibility by wireless communication and modular control loops allows rapid adjustments to dynamic workflows.

Scalability by the integration of SCADA and IoT protocols supports scaling for more complex systems.

User-friendly monitoring by HMI-MCPRS offers intuitive interfaces for local and remote users to monitor and control system operations effectively.

### 4.3. Real-Time Control of MPS Assisted by MCPRS

The real-time control of the modular production system (MPS), assisted by the MCPRS, relies on advanced perception, computation, and actuation strategies to achieve precise picking, placing, and transporting tasks. This integration of robotic manipulation and mobile vision-based control ensures high reliability for P/R/S operations. The assisting P/R/S technology for MPS consists of one dynamic robotic system: MCPRS, which is used in picking, placing, and transporting the WP. Based on the inverse kinematic control, the remote PC-HMI-MCPRS calculates the order for the Cyton 1500 RM for parking and positioning related to pick-up and rough plating operations. Based on the method of moments of the image, the remote PC-HMI-MCPRS calculates the command for fine positioning the end-effector of the Cyton RM for picking and placing the WP.

**Figure 18:**
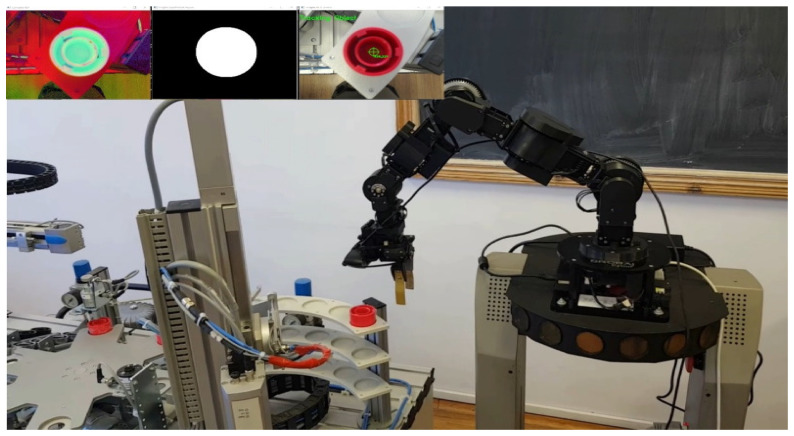
MVSS-based Cyton RM control for WP’s picking up from WS4. In the top-left medallion is WP’s detection with the following steps: conversion from RGB to HSV; image segmentation after the color has been found between the HSV limits and the shape corresponding to the object has been found; object color and shape have been found and is being tracked.

**Figure 19:**
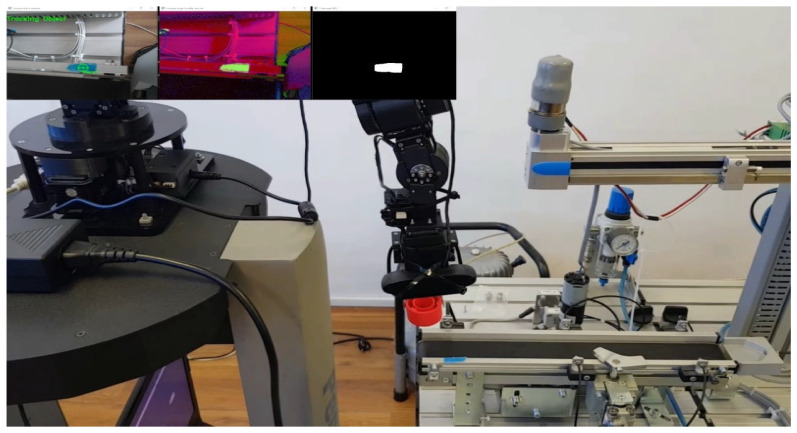
MVSS-based Cyton RM for WP being placed on WS1. In the top-left medallion is reference point detection with the following steps: conversion from RGB to HSV; image segmentation after the color has been found between the HSV limits and the shape corresponding to the reference has been found; reference object color and shape have been found and is being tracked.

- Picking operations, detection, and precision control: The remote or local PC-HMI-MCPRS calculates positioning commands for the Cyton 1500 robotic manipulator (RM). These commands guide the RM for initial positioning for pickup operations at WS4. In Figure 18, the MVSS steps for WP detection are shown when taking over from WS4. On the upper left side in Figure 18 is vision-based detection by MVSS. Detection utilizes RGB-to-HSV color model conversion for robustness under varying lighting conditions. HSV better handles light changes compared to RGB, crucial during transitions between natural and artificial lighting. Object shape and position are determined using the Ramer–Douglas–Peucker algorithm that simplifies the object contour for shape analysis. Canny Edge Detection identifies the edges of the object for precise contouring. Centroid tracking employs the method of image moments, ensuring efficient 2D tracking. The process is robust for circular objects, consistently identifying their centroid within acceptable error limits. All of this was implemented using the OpenCV libraries [37]. Finally, in the image on the top right of the medallion in Figure 18, the object is tracked, meaning the target has been identified, if both the color and shape conditions were simultaneously met [13].
- Vision-based detection, alignment reference point and placing operations: On the upper left side in Figure 19 is WS1 detection and alignment reference point detection. The WS1 placement relies on detecting a rectangular reference point, contrasting circular object detection at WS4. This step ensures accurate alignment for reprocessing or scrapping. Fine Positioning with MVSS: like positional refinement, this is based on real-time feedback from MVSS. Adjustments in the end-effector positioning minimize error before placement.

Experimental results: By addressing the below challenges, the MPS assisted by MCPRS can achieve higher reliability, improved efficiency, and precision closer to industrial standards.

- Fine Positioning with MVSS

Purpose: Positional refinement based on real-time feedback from MVSS ensures precision in WP picking and placing tasks.

WP Picking: Real-time vision data from the MVSS guide the robotic manipulator to adjust its end-effector position and orientation dynamically. This minimizes positional errors during the grasping process.

WP Placing: Before placing the WP, the MVSS feedback helps fine-tune the end-effector’s alignment with the target, reducing error margins and ensuring accurate placement.

- Integrated Technologies for Efficiency and Precision

Inverse Kinematics: Enables precise positioning and orientation control of the Cyton 1500 robotic manipulator’s 7-DOF arm, accommodating complex movements and angular constraints.

Real-Time Vision-Based Control: Provides continuous feedback on the WP’s position, compensating for environmental and system uncertainties.

Synchronized Trajectory Planning: Harmonizes movements between the MCPRS and the MPS for smooth and efficient operations.

- Trajectory Optimization and Performance

**Figure 20:**
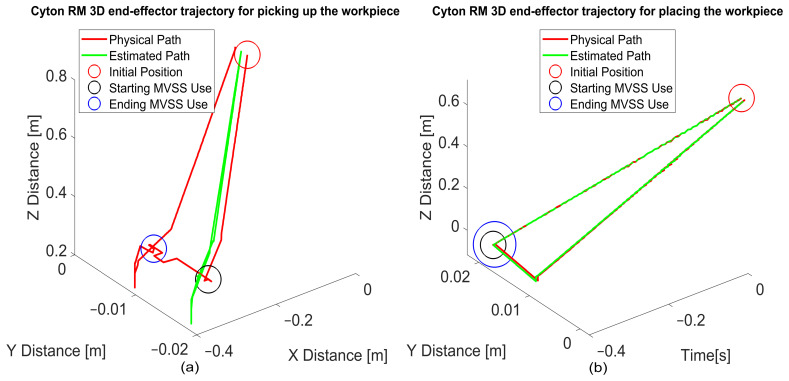
Estimated (desired) and physical (real) 3D trajectories of Cyton RM for: (**a**) WP’s picking from WS4; (**b**) WP’s placing on WS1.

3D Trajectory Planning:

Picking Tasks: Figure 20a illustrates the full trajectory, with waypoints including home, scanning, above object, picking, and return to home.

**Placing Tasks:** Figure 20b shows a similarly structured trajectory, focusing on precise placement.

- **Deviation Observations:**

For WP picking, the primary deviation occurs along the Y-axis, with a peak of 10 mm that stabilizes during the task.

During WP placing, deviations are significantly reduced due to enhanced alignment controls.

**Figure 21:**
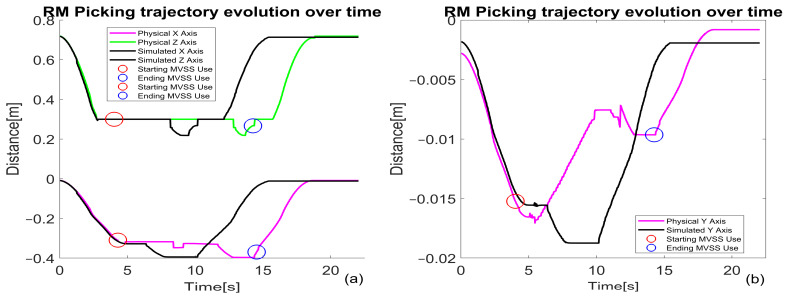
Simulated and physical real trajectories evolution over time for (**a**) X and Z axis and (**b**) Y axis for picking the workpiece.

**Figure 22:**
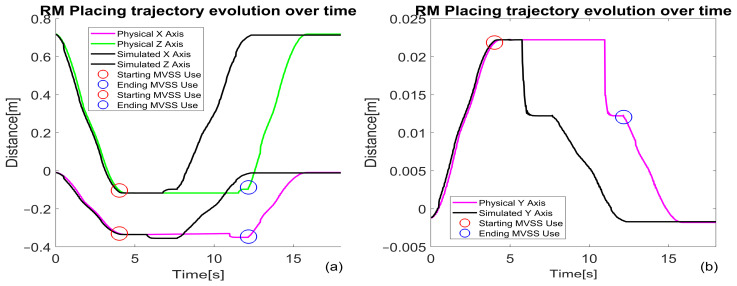
Simulated and physical real trajectories evolution over time for (**a**) X and Z axis and (**b**) Y axis for placing the workpiece.

- **Efficiency Analysis** (Figure 21 and Figure 22):

Picking tasks exhibit a cycle time of over 10 s, while placing tasks average around 8 s. Time discrepancies are primarily due to higher deviations during initial alignment challenges in picking.

- **Trajectory Precision through Real-Time Feedback**

Real-time visual feedback from the MVSS dynamically corrects trajectory deviations during operations.

Figure 22a,b illustrates how these corrections occur over time, ensuring precision in both picking and placing tasks.

- **Transport Operations with PeopleBot WMR**

**Control Method:** The PeopleBot WMR uses Digital Twin-Time Trajectory Sliding Mode Control (DT-TTSMC) for trajectory management. Position feedback facilitates real-time error correction, enhancing transport accuracy.

**Figure 23:**
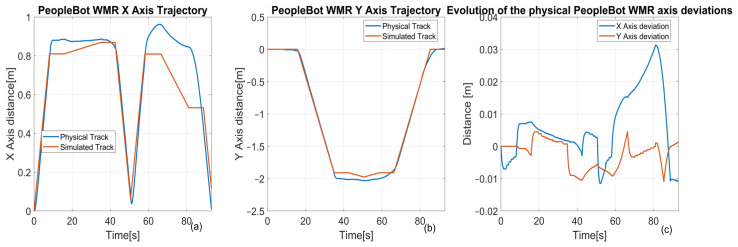
MCPRS’s real-time and simulated in MobileSim trajectories; (**a**) along X-axis, (**b**) along Y-axis, and (**c**) MCPRS’s X and Y axis deviations.

- **Performance Analysis** (Figure 23): X-axis deviations reach up to 10 mm (Figure 23a,c). Y-axis deviations peak at 5 mm (Figure 23b,c). These results demonstrate high alignment accuracy during transport, critical for reliable task execution.
- Challenges and Improvement Opportunities:

**System Limitations:** The observed tracking errors (10 mm on the X-axis and 5 mm on the Y-axis) stem from the research-grade nature of the MPS stations and MCPRS components. These laboratory systems lack the precision of industrial-grade equipment.

**Potential Enhancements:** Improved vision algorithms to bolster object detection and environmental adaptability. Enhanced lighting adaptability for consistent visual performance in varying conditions. Advanced trajectory correction mechanisms to minimize deviations further and reduce cycle times.

## 5. Discussion

A comprehensive system architecture for the cloud/VPN-based remote control of MPS (four-workstation modular production system) assisted by a MCPRS is presented. The remote control of the described system integrates advanced IoT, cloud, and VPN technologies with hierarchical automation for seamless operation and monitoring. It enables users to interact with, control, and optimize production and handling tasks remotely through a robust framework.

The benefits of approaching such a production system include the following:

- Remote control and flexibility through the cloud/VPN system. Users can control MPS and MCPRS by AR interfaces for task management. The VPN ensures secure access, while OPC-UA facilitates data sharing with the cloud platform.
- Enhanced workflow optimization by using SHPN simulation allows for systematic modeling of both continuous and discrete processes, helping to optimize task execution and prevent bottlenecks in the system.
- Advanced communication infrastructure by using Profibus DP, Profinet, Ethernet, and OPC-UA ensures real-time, reliable communication across the system, with seamless integration between the production line, robotic systems, and the cloud.
- Interoperability and future scalability with OPC-UA, LAN Profinet, and LAN Ethernet, the system is future-proof, allowing for the easy integration of additional devices, sensors, or workstations as needed.
- Real-time task planning and visualization by planning tasks, simulating workflows, and visualizing the system in real time through AR and VR interfaces, enabling quick decisions and optimizations.
- Enhanced flexibility by using SHPN, TPN, and CPN models allows for flexible task execution, supporting both discrete (stationary WSs) and continuous (MCPRS) operations.
- Fault detection and reprocessing with MCPRS assist in reprocessing defective WPs, reducing downtime and improving overall production efficiency. Visual feedback through MVSS allows the MCPRS to autonomously handle quality control tasks.
- Cloud-based control through OPC-UA and WAN Ethernet, the system can be monitored and controlled remotely, offering cloud-based SCADA/HMI interfaces for enhanced accessibility and control from any location.

To assess the effectiveness of a cloud/VPN-based remote-control system for MPS assisted by MCPRS utilizing the DT approach, a systematic evaluation strategy is required. This strategy must consider performance, functionality, reliability, and user satisfaction. Below are the methods for evaluation:

- Measure the percentage of tasks successfully completed without interruptions or errors, such as processing, reprocessing, and scrapping workflows. Evaluate the synchronization between the MPS and MCPRS. Use metrics like delay in command execution or deviations between DT simulations and real-world actions.
- Test the latency of the cloud/VPN connection, particularly for critical operations requiring real-time feedback (task planning with AR or reprocessing tasks). Monitor the availability of the system components (PLCs, embedded computer, MCPRS, WSs and DT) under varying operational loads. Measure the number of WPs processed, reprocessed, or scrapped per unit of time.
- Simulate scenarios with varying task complexities and multiple users remotely access the system to test scalability. Evaluate how well the system adapts to changes in production requirements, including the addition of new tasks or equipment.
- Conduct surveys or interviews with remote and local users to understand their experience with AR-based task planning and VR visualizations. Evaluate HMIs in terms of intuitiveness, error rates, and ease of navigation. Measure the time required for new users to learn and operate the system efficiently.
- Count system failures, communication errors, or downtime incidents over a defined period. Conduct penetration testing to evaluate the resilience of the Cloud/VPN infrastructure against cyber threats.
- Compare the DT simulation outcomes with real-world results. Metrics include deviation in task execution times or movements of the MCPRS. Assess the accuracy and usefulness of AI (machine learning)-based maintenance alerts or process optimizations.
- Monitor the energy efficiency of the MPS and MCPRS during operations. Calculate the costs associated with remote operations and compare them to traditional on-site methods.
- Use SHPNs to simulate different production scenarios and validate system responses. Leverage SCADA systems to display and analyze key performance indicators in real time. Compare the system’s performance against industry standards or similar setups.

Designing HMIs for the Cloud/VPN-based remote control of a MPS assisted by a MCPRS with a DT approach demands careful consideration of user experience, usability, and user feedback mechanisms to ensure effective operation in Industry 4.0 and 5.0 environments by the following:

- Ensure HMIs provide intuitive, real-time visual feedback about the status of the MPS and MCPRS (WP location, task progress, and errors). Include 3D models and AR/VR representations linked to the DT to visualize task planning and execution interactively. Implement customizable dashboards, allowing users to tailor views based on their roles. Use touch-based or voice-controlled interfaces for ease of operation, especially for mobile or wearable devices. Maintain consistency in layout, color schemes, and symbols across various control panels for different systems (MCPRS control vs. MPS task planning). Follow established user interface design guidelines, such as those from ISO standards for industrial interfaces. Include built-in mechanisms for guiding users to avoid common errors (confirmations for critical actions, visual warnings for system constraints). Provide contextual troubleshooting suggestions based on detected issues. Provide contextual troubleshooting suggestions based on detected issues.
- Design hierarchical menus logically, ensuring quick access to critical functions such as emergency stops or manual overrides. Enable multi-layer zoom for detailed and high-level views of the entire MPS and MCPRS workflows. Use automated task suggestions and assistive AI tools to streamline repetitive or complex operations. Offer drag-and-drop task scheduling in AR/VR environments for task planning. Provide auditory, haptic, or visual feedback to confirm actions, such as successful execution of reprocessing and scrapping command or MCPRS movement. Ensure HMIs are accessible to diverse users, including features like multilingual support, adjustable text sizes, and high-contrast modes.
- Include in-app feedback forms or direct links to reporting issues or requesting new features. Implement a feedback loop where operators receive responses to their suggestions or concerns. Track user interaction metrics, average task time, and error rates to identify usability bottlenecks. Regularly test HMIs with end-users during design and after deployment.
- Enable users to manipulate 3D models in AR to visualize and modify workflows dynamically. Allow operators to walk through virtual environments to practice controlling MPS and MCPRS workflows or diagnose issues remotely.
- Log errors to identify areas where HMI complexity may lead to mistakes. Adjust workflows to be more user-friendly based on error patterns. Provide interactive tutorials or simulation-based training directly within HMIs, utilizing DT visualizations. Regularly assess how new operators adapt to the system and refine features to reduce the learning curve.

To examine potential cybersecurity threats and mitigation techniques for MPS assisted by MCPRS with the DT approach, it is essential to identify and assess risks associated with each layer of the system and implement robust countermeasures.

- Mitigate unauthorized access to sensitive production data stored in the cloud. VPN vulnerabilities, such as default configurations or weak encryption, lead to eavesdropping or unauthorized access. Overwhelm the cloud service with traffic to disrupt operations. Attackers gain higher access levels in the cloud environment. Conduct regular vulnerability scans and penetration tests on the VPN and cloud systems.
- Intercept data between physical and virtual digital twins. Introducing false data to the DT for incorrect task planning or decision-making. Modify task planning or simulations in AR/VR. Monitor the consistency between physical and virtual digital twins with hash-based verification. Use real-time validation for AR/VR interactions to prevent discrepancies.
- Intercept traffic on OPC UA or Profinet and Ethernet networks. Re-send valid commands to disrupt synchronized operations. Encrypt MPS control data to halt operations. Deploy intrusion detection systems for LAN/WAN traffic. Conduct protocol fuzzing for OPC UA and Profinet to identify weaknesses.
- Exploit interfaces to control MCPRS movements or manipulations. Tamper with MVSS inputs to misdirect MCPRS operations. Target vulnerabilities in WMR or RM software. Validate commands with digital signatures. Use redundant sensor fusion to detect anomalies in localization.
- Enforce end-to-end encryption data transmission. Implement multi-factor authentication for cloud and VPN access. Use zero-trust architecture for access control, ensuring only verified users/devices can connect. Regularly apply security patches to cloud platforms and VPN clients.
- Use encrypted data streams between physical and digital twins. Deploy blockchain for immutable record-keeping of DT transactions. Implement role-based access control for AR/VR task planning. Use AI anomaly detection to identify malicious activity in DT simulations.
- Secure RM and WMR firmware with secure boot mechanisms. Introduce runtime integrity checks for MCPRS operations. Use geofencing policies to limit MCPRS operation to specific areas.
- Develop and enforce cybersecurity policies for employees and contractors. Deploy endpoint detection and response tools across WSs and PLCs. Establish an incident response plan and perform regular cybersecurity drills.
- Ensure the implemented cybersecurity measures are effective. Conduct periodic audits and risk assessments. Use penetration testing and red teaming to simulate attacks. Track key performance indicators, such as the number of blocked intrusion attempts or time to detect/respond to threats. Regularly review and update the system against emerging threats.

To compare our proposed approach with others in the field, we undertook a literature review following the PRISMA guidelines or Systematic Literature Review (SLR) mapping techniques. Thus, we structured and completed the content of the paper and references based on the following actions:

- We conducted a comprehensive analysis of existing research on digital twin (DT) technology, with a special focus on its integration into modular manufacturing systems (MPS) and mobile cyber–physical robotic systems (MCPRS).
- We highlighted the limited studies on hybrid DT systems that combine modular manufacturing lines and mobile robotic platforms, especially those that use AR/VR and SHPN modeling. We selected two methods for comparison, which are briefly presented below.

We believe that the proposed research fills these gaps by developing a SHPN-based DT approach for MPS and MCPRS integration, leveraging AR/VR for improved user interaction and real-time task planning. It reiterates the novelty of the proposed DT framework and its contributions to academia and industry.

To compare recent literature methods with the proposed DT approach for tracking errors in a MPS assisted by a MCPRS, the following structure highlights three key approaches, focusing on tracking error metrics:

- **Our proposed method**: DT-based MPS and MCPRS integration.

**Key Features:**

**SHPN Simulation as VR**: Combines TPN for MPS processes and CPN for MCPRS movements.

**Node-RED as AR for Task Planning**: Real-time task visualization and planning.

**Real-Time Synchronization**: Ensures seamless coordination between MPS and MCPRS subsystems through hybrid modeling.

**Tracking Error**: Minimal error due to robust synchronization of discrete and continuous tasks using SHPN, validated by experimental results with errors ≤5 mm for robotic manipulator positioning and ≤10 mm for mobile platform movement. However, these relatively large tracking errors are because both the MPS stations and the MCPRS components are laboratory research equipment, which is less accurate, and not precision industrial ones.

Comparison with two other literature methods.

- **Method 1:** Dynamic Job-Shop scheduling with Cyber–Physical Systems (CPS) integration [38,39,40]:

**Description:** Combines CPS with reinforcement learning to optimize job-shop scheduling in modular production systems. Uses machine learning for adaptive control but lacks real-time visual task planning.

**Strengths**: Achieves dynamic task allocation and reduced delays in high-demand scenarios.

**Weaknesses:** Does not explicitly handle robotic manipulators or mobile platforms. Higher tracking error observed for dynamic workpiece routing due to indirect synchronization methods (~15 mm error for path-following tasks).

- **Method 2:** IoT-enabled predictive maintenance in smart manufacturing [41,42]:

**Description:** Integrates IoT sensors with predictive maintenance to minimize downtime in modular production systems. Utilizes digital twins for fault detection but does not include AR/VR for interactive task planning.

**Strengths:** Effective for fault prediction and diagnosis. Reduces system downtime significantly.

**Weaknesses:** Limited real-time task synchronization between production and mobile robotic systems. Tracking errors for fault location and correction averaged ~14 mm, primarily in robotic arm operations due to a delay in sensor feedback loops.

## 6. DT-Literature Review Using PRISMA and the Systematic Literature Review

In this section, there is a revised literature review based on PRISMA guidelines and the Systematic Literature Review (SLR) approach in relation to which the content of the paper and the chosen references were structured effectively. The review provides a clear flow and focuses on the topic of DT applications involved in remote control of MPS assisted by MCPRS.

- **Introduction.** The adoption of DT technology in manufacturing and robotics is pivotal in advancing Industry 4.0 and transitioning to Industry 5.0 paradigms. This literature review systematically analyzes the state-of-the-art methodologies and applications in DT for MPS assisted by MCPRS. The review follows PRISMA guidelines, ensuring a structured and comprehensive assessment of the existing literature.
- **Methodology.** The literature was reviewed using the PRISMA framework included the following:

**Identification**: Searches were conducted in databases such as IEEE Xplore, ScienceDirect, MDPI, and Springer for publications between 2015 and 2023 using keywords: “Digital Twin”, “Modular Production System”, “Mobile Cyber–Physical Systems”, “Industry 4.0”, and “Industry 5.0”.

**Screening**: Articles were filtered based on relevance, focusing on DT applications in manufacturing, robotic systems, and control frameworks.

**Eligibility**: Only peer-reviewed articles discussing MPS, MCPRS, or related control architectures were included.

**Inclusion**: The final reference selection consisted of 42 articles that specifically address DT in manufacturing and robotics.

- **Key Themes and Findings**

**Digital Twin Architectures:** Segovia et al. (2022) presented a DT architecture integrating real-time data from sensors and simulations for enhanced control in manufacturing. Similarly, Martinez et al. (2021) utilized the automation pyramid to build DTs for educational manufacturing systems, emphasizing hierarchical control. Bamunuarachchi et al. (2021) explored cloud-edge collaboration in DT implementation, highlighting the role of the Industrial Internet of Things (IIoT).

**DT in Modular Production Systems:** Mincă et al. (2022) proposed a DT framework for flexible assembly lines integrating robotic manipulators and visual servoing systems. This aligns with Moiceanu and Paraschiv (2022), who conducted a bibliometric analysis of DT applications in smart manufacturing, emphasizing modularity and flexibility.

**DT for Mobile Cyber–Physical Robotic Systems:** Simion et al. (2022) demonstrated the control of autonomous MCPRS through mobile visual servoing, integrating DT for process monitoring. Gallala et al. (2022) applied DT to human–robot interactions, leveraging Industry 4.0 technologies for task synchronization. Stączek et al. (2021) used DT to optimize logistics in production environments with autonomous mobile robots.

**Integration of DT with Control Architectures:** Bradley and Atkins (2015) explored optimization techniques for cyber–physical vehicle systems, forming the foundation for DT-based control in robotic platforms. Filipescu et al. (2023) highlighted the synchronization of hybrid Petri nets (SHPN) in DT systems, combining Timed and Continuous Petri Nets for precise control of MPS and MCPRS.

**Challenges and Future Directions:** Despite advancements, challenges remain in ensuring real-time synchronization between physical and virtual systems. Zhang et al. (2022) and Liu et al. (2022) addressed deep learning integration for predictive maintenance in DT, emphasizing the potential of augmented reality (AR) for task planning. Li et al. (2023) proposed remote monitoring platforms for DT in industrial applications, advocating cloud-based solutions for scalability.

**Comparative Analysis**: Table 1 provides a comparative analysis of key studies.

**Final remarks.** The systematic review reveals that DT implementation in MPS and MCPRS offers significant improvements in flexibility, precision, and scalability. However, further research is needed to address real-time synchronization and integration challenges, particularly in the context of Industry 5.0, where human-centric and sustainable manufacturing systems are emphasized.

## 7. Conclusions

To more clearly articulate the contribution and novelty of this approach, we need to refine the objectives formulated in the Introduction section.

- Develop a DT framework integrating MPS and MCPRS for enhanced task planning, monitoring, and synchronization.
- Implement a hybrid SHPN model to ensure precise synchronization of discrete MPS operations and continuous MCPRS movements.
- Demonstrate the application of AR and VR technologies for real-time task planning, error handling, and operational insight in a modular production setting.
- Validate the system’s capability to reallocate tasks dynamically, including transporting defective workpieces from WS4 to WS1 for reprocessing or scrapping.
- Provide a robust teaching and research platform for Industry 4.0 and 5.0 applications, emphasizing real-time control, predictive analytics, and IoT-enabled manufacturing.

This research presents a novel integration of the DT approach into a processing technology framework involving a MPS assisted by a MCPRS. The unique aspects and contributions of the approach include:

- Innovative DT framework for hybrid systems: The proposed system employs a DT framework that bridges real-world hardware with virtual representations using augmented reality (AR) and virtual reality (VR). This integration enhances real-time task planning, monitoring, and synchronization of operations between MPS and MCPRS.
- Advanced hardware architecture: The architecture features a line-shaped MPS with four workstations (WS1 to WS4) and a MCPRS equipped with a 2DW/1FW-WMR, a 7-DOF RM, and MVSS mounted on the RM’s end effector. This configuration enables efficient workpiece handling, transport, and processing, including reprocessing or scrapping operations.
- Hierarchical control system: The MPS control system integrates four workstation PLCs connected via Profibus DP to a central PLC, ensuring modularity and interoperability. The system extends connectivity with LAN Profinet, LAN Ethernet, and WAN Ethernet using OPC-UA, facilitating seamless local and cloud-based operations.
- SHPN for system coordination: A novel use of SHPN, combining Timed Petri Nets (TPN) for discrete MPS processes and Continuous Petri Nets (CPN) for MCPRS movement, provides a synchronized virtual representation of tasks. The SHPN simulation enables precise synchronization between discrete and continuous system dynamics, validated through real-time operational data.
- Enhanced user interaction with AR and VR: The AR-based task planning interface allows users to interact with production workflows in real time, visualize task schedules, and adapt to errors dynamically. VR-based simulation using SHPN improves transparency, offering an intuitive and immersive environment for understanding and optimizing system behavior.
- The effectiveness of the Cloud/VPN-based remote control of an MPS and MCPRS can be systematically assessed by focusing on functional, performance, reliability, and user-centric metrics. Continuous monitoring and iterative improvement cycles, informed by collected data and feedback, ensure the system remains robust and aligned with Industry 4.0 and 5.0 objectives.
- An effective HMI design ensures seamless integration of cloud/VPN-based remote control, DT visualizations, and MCPRS interactions, offering a balance of functionality and ease of use. Incorporating usability and user feedback mechanisms allows the system to evolve continuously, meeting the dynamic demands of Industry 4.0 and 5.0.
- By integrating strategies for cybersecurity threats and mitigation techniques the cloud/VPN-based remote-control system with DT technology can remain robust, resilient, sustainable, and secure against a wide array of cybersecurity threats (demand of Industry 5.0).

The integration of these elements highlights the novelty of the proposed DT approach, making it a significant contribution to both academia and industry, advancing the state-of-the-art in manufacturing systems.

## Figures and Tables

**Table 1:** Comparative analysis of key studies.

Study	Focus Area	Key Contributions
Mincă et al. (2022)	MPS and robotic systems	Flexible assembly with DT integration
Simion et al. (2022)	MCPRS	Mobile visual servoing for autonomous tasks
Segovia et al. (2022)	DT architectures	Real-time data integration and control
Zhang et al. (2022)	Predictive maintenance	Deep learning-enhanced DT applications
Filipescu et al. (2023)	SHPN for DT	Task synchronization using hybrid Petri nets

## Data Availability

Data are contained within the article.
